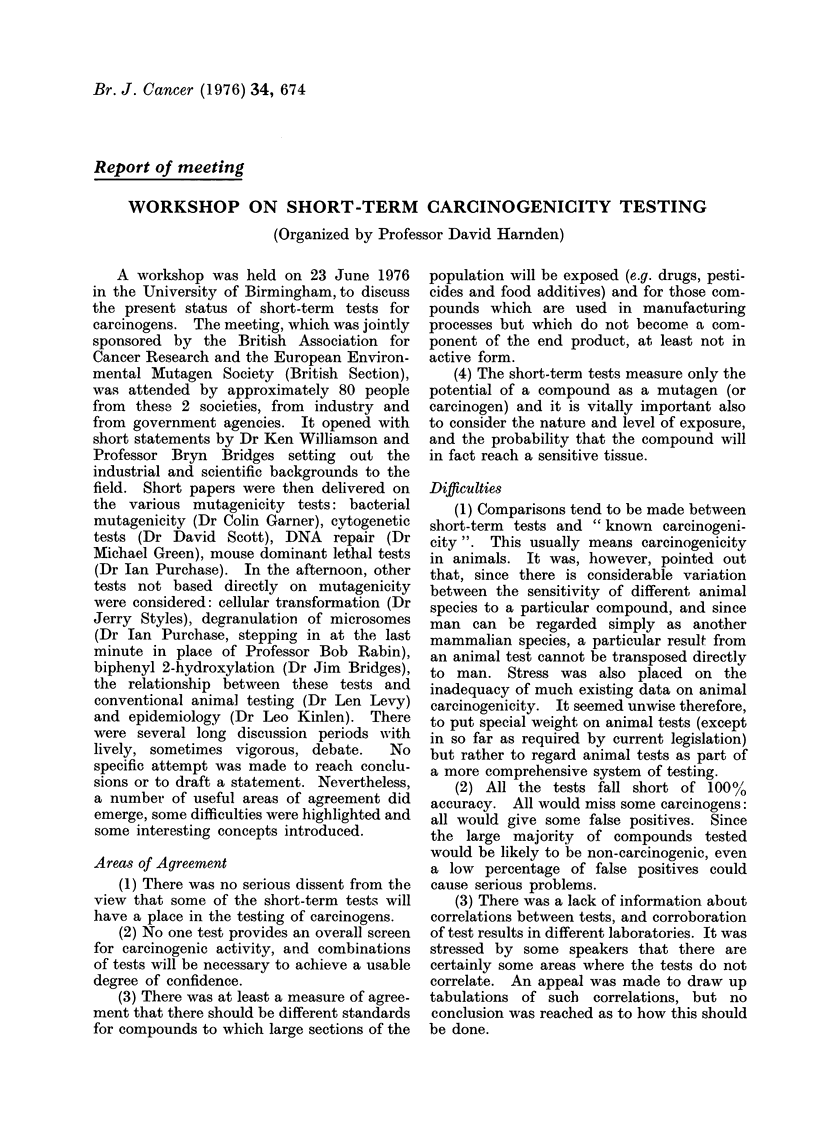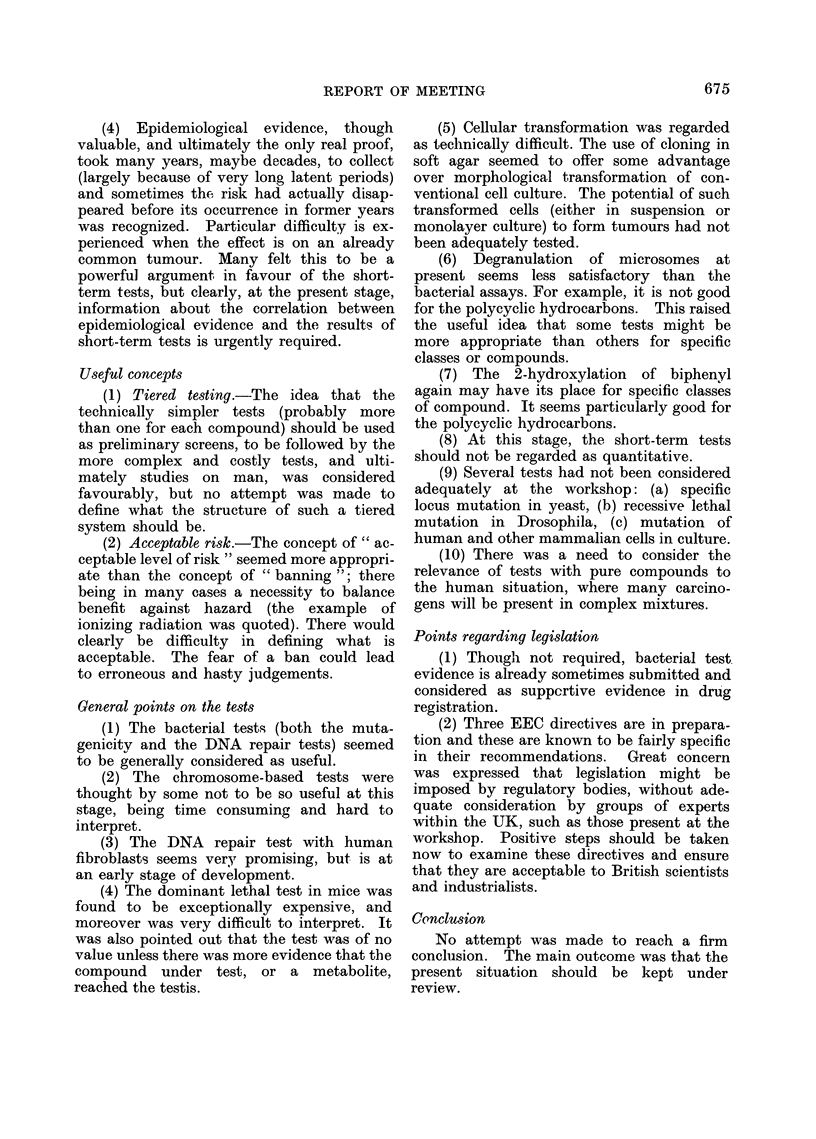# Workshop on Short-term Carcinogenicity Testing

**Published:** 1976-12

**Authors:** 


Br. J. Cancer (1976) 34, 674

*Report of meeting*

### WORKSHOP ON SHORT-TERM CARCINOGENICITY TESTING

(Organized by Professor David Harnden)

A workshop was held on 23 June 1976 in the University of Birmingham, to discuss the present status of short-term tests for carcinogens. The meeting, which was jointly sponsored by the British Association for Cancer Research and the European Environmental Mutagen Society (British Section), was attended by approximately 80 people from these 2 societies, from industry and from government agencies. It opened with short statements by Dr Ken Williamson and Professor Bryn Bridges setting out the industrial and scientific backgrounds to the field. Short papers were then delivered on the various mutagenicity tests: bacterial mutagenicity (Dr Colin Garner), cytogenetic tests (Dr David Scott), DNA repair (Dr Michael Green), mouse dominant lethal tests (Dr Ian Purchase). In the afternoon, other tests not based directly on mutagenicity were considered: cellular transformation (Dr Jerry Styles), degranulation of microsomes (Dr Ian Purchase, stepping in at the last minute in place of Professor Bob Rabin), biphenyl 2-hydroxylation (Dr Jim Bridges), the relationship between these tests and conventional animal testing (Dr Len Levy) and epidemiology (Dr Leo Kinlen). There were several long discussion periods with lively, sometimes vigorous, debate. No specific attempt was made to reach conclusions or to draft a statement. Nevertheless, a number of useful areas of agreement did emerge, some difficulties were highlighted and some interesting concepts introduced.

*Areas of Agreement*

(1) There was no serious dissent from the view that some of the short-term tests will have a place in the testing of carcinogens.

(2) No one test provides an overall screen for carcinogenic activity, and combinations of tests will be necessary to achieve a usable degree of confidence.

(3) There was at least a measure of agreement that there should be different standards for compounds to which large sections of the population will be exposed (*e.g.* drugs, pesticides and food additives) and for those compounds which are used in manufacturing processes but which do not become a component of the end product, at least not in active form.

(4) The short-term tests measure only the potential of a compound as a mutagen (or carcinogen) and it is vitally important also to consider the nature and level of exposure, and the probability that the compound will in fact reach a sensitive tissue.

*Difficulties*

(1) Comparisons tend to be made between short-term tests and " known carcinogenicity ". This usually means carcinogenicity in animals. It was, however, pointed out that, since there is considerable variation between the sensitivity of different animal species to a particular compound, and since man can be regarded simply as another mammalian species, a particular result from an animal test cannot be transposed directly to man. Stress was also placed on the inadequacy of much existing data on animal carcinogenicity. It seemed unwise therefore, to put special weight on animal tests (except in so far as required by current legislation) but rather to regard animal tests as part of a more comprehensive system of testing.

(2) All the tests fall short of 100% accuracy. All would miss some carcinogens: all would give some false positives. Since the large majority of compounds tested would be likely to be non-carcinogenic, even a low percentage of false positives could cause serious problems.

(3) There was a lack of information about correlations between tests, and corroboration of test results in different laboratories. It was stressed by some speakers that there are certainly some areas where the tests do not correlate. An appeal was made to draw up tabulations of such correlations, but no conclusion was reached as to how this should be done.

(4) Epidemiological evidence, though valuable, and ultimately the only real proof, took many years, maybe decades, to collect (largely because of very long latent periods) and sometimes the risk had actually disappeared before its occurrence in former years was recognized. Particular difficulty is experienced when the effect is on an already common tumour. Many felt this to be a powerful argument in favour of the short-term tests, but clearly, at the present stage, information about the correlation between epidemiological evidence and the results of short-term tests is urgently required.

*Useful concepts*

(1) *Tiered testing.*—The idea that the technically simpler tests (probably more than one for each compound) should be used as preliminary screens, to be followed by the more complex and costly tests, and ultimately studies on man, was considered favourably, but no attempt was made to define what the structure of such a tiered system should be.

(2) *Acceptable risk.*—The concept of " acceptable level of risk " seemed more appropriate than the concept of " banning "; there being in many cases a necessity to balance benefit against hazard (the example of ionizing radiation was quoted). There would clearly be difficulty in defining what is acceptable. The fear of a ban could lead to erroneous and hasty judgements.

*General points on the tests*

(1) The bacterial tests (both the mutagenicity and the DNA repair tests) seemed to be generally considered as useful.

(2) The chromosome-based tests were thought by some not to be so useful at this stage, being time consuming and hard to interpret.

(3) The DNA repair test with human fibroblasts seems very promising, but is at an early stage of development.

(4) The dominant lethal test in mice was found to be exceptionally expensive, and moreover was very difficult to interpret. It was also pointed out that the test was of no value unless there was more evidence that the compound under test, or a metabolite, reached the testis.

(5) Cellular transformation was regarded as technically difficult. The use of cloning in soft agar seemed to offer some advantage over morphological transformation of conventional cell culture. The potential of such transformed cells (either in suspension or monolayer culture) to form tumours had not been adequately tested.

(6) Degranulation of microsomes at present seems less satisfactory than the bacterial assays. For example, it is not good for the polycyclic hydrocarbons. This raised the useful idea that some tests might be more appropriate than others for specific classes or compounds.

(7) The 2-hydroxylation of biphenyl again may have its place for specific classes of compound. It seems particularly good for the polycyclic hydrocarbons.

(8) At this stage, the short-term tests should not be regarded as quantitative.

(9) Several tests had not been considered adequately at the workshop: (a) specific locus mutation in yeast, (b) recessive lethal mutation in Drosophila, (c) mutation of human and other mammalian cells in culture.

(10) There was a need to consider the relevance of tests with pure compounds to the human situation, where many carcinogens will be present in complex mixtures.

*Points regarding legislation*

(1) Though not required, bacterial test evidence is already sometimes submitted and considered as supportive evidence in drug registration.

(2) Three EEC directives are in preparation and these are known to be fairly specific in their recommendations. Great concern was expressed that legislation might be imposed by regulatory bodies, without adequate consideration by groups of experts within the UK, such as those present at the workshop. Positive steps should be taken now to examine these directives and ensure that they are acceptable to British scientists and industrialists.

*Conclusion*

No attempt was made to reach a firm conclusion. The main outcome was that the present situation should be kept under review.